# How Do Radiologists Currently Monitor AI in Radiology and What Challenges Do They Face? An Interview Study and Qualitative Analysis

**DOI:** 10.1007/s10278-025-01493-8

**Published:** 2025-04-08

**Authors:** Jamie Chow, Ryan Lee, Honghan Wu

**Affiliations:** 1 Institute of Health Informatics, University College London, London, UK; 2 Sidney Kimmel Medical College at Jefferson Health, Philadelphia, PA USA

**Keywords:** Radiologist, Artificial intelligence, Quality assurance, Audit, Monitoring, Surveillance

## Abstract

Artificial intelligence (AI) in radiology is becoming increasingly prevalent; however, there is not a clear picture of how AI is being monitored today and how this should practically be done given the inherent risk of AI model performance degradation over time. This research investigates current practices and what difficulties radiologists face in monitoring AI. Semi-structured virtual interviews were conducted with 6 USA and 10 Europe-based radiologists. The interviews were automatically transcribed and underwent thematic analysis. The findings suggest that AI monitoring in radiology is still relatively nascent as most of the AI projects had not yet progressed into a fully live clinical deployment. The most common method of monitoring involved a manual process of retrospectively comparing the AI results against the radiology report. Automated and statistical methods of monitoring were much less common. The biggest challenges are a lack of resources to support AI monitoring and uncertainty about how to create a robust and scalable process of monitoring the breadth and variety of radiology AI applications available. There is currently a lack of practical guidelines on how to monitor AI which has led to a variety of approaches being proposed from both healthcare providers and vendors. An ensemble of mixed methods is recommended to monitor AI across multiple domains and metrics. This will be enabled by appropriate allocation of resources and the formation of robust and diverse multidisciplinary AI governance groups.

## Background

As of December 2024, there are 1016 FDA-cleared artificial intelligence (AI)/machine learning enabled medical devices [1] of which 777 (76%) belongs to radiology. This number is not inclusive of medical devices regulatory cleared outside of the USA or the growing number of in-house developed AI applications.

The growing adoption of AI in radiology [2] is primarily driven by their potential to improve efficiency and improve patient outcomes [3, 4]. The 2023 UK Clinical Radiology Workforce census reports that 54% of trusts or health boards are currently using AI tools in clinical use [5] AI models are, however, prone to performance degradation over time [6, 7]. A change in input variable may result in data shift [8], for example, a change in the scanning protocol or scanning equipment from which the AI model had initially been trained on may adversely affect AI model performance. An unexpected change in local disease prevalence, such as during the COVID-19 pandemic [9], may also affect the predictive performance of disease-classifying AI applications.

Potential harm may arise from inherent biases within AI applications which may in turn exacerbate health inequality [10]. A review [11] into the geographic distribution of US cohorts used to train deep learning algorithms found that most of them were represented by 3 states: California, Massachusetts, and New York. AI models that are trained on a non-representative dataset may not generalize well to the local environment in which they will ultimately be deployed. The lack of transparency around the patient datasets used to train and validate commercial AI applications [12, 13] compounds this problem and can present a challenge for AI adopters looking to select the most suitable AI application for their local environment and patient population. This is particularly important in the USA where Sect. 1557 of the Affordable Care Act [14] makes it unlawful for healthcare providers receiving Federal financial assistance, to discriminate against individuals based on their race, color, national origin, sex, age, or disability.

In order to mitigate against the above issues, it is recommended that local validation of AI models is performed before deployment, and then a subsequent process established to monitor their performance over time [15–17]. Andersen et al. [18] recently conducted a scoping review of how the performance of clinical AI applications is monitored and found a lack of guidance for the practical implementation of AI performance monitoring. This was highlighted by the over-representation of opinion papers/narrative reviews and simulation studies as opposed to clinical trials and real-world implementation studies.

### Current Frameworks and AI Monitoring Methodologies

Raji et al. [19] use concepts and auditing procedures from other industries such as failure modes and effects analysis. The resultant “SMACTR” framework is composed of five distinct stages: Scoping, Mapping, Artifact Collection, Testing, and Reflection. Liu et al. [20] adopted this framework for AI applications in medicine which developers and AI adopters can use to validate, monitor, and review errors.

Mahmood et al. [21] also reference the use of established quality assurance practices in the example of the Mammography Quality Standards Act (MQSA) [22] in the USA. This is a program designed to assure continued quality control of mammography by reviewing the equipment, personnel, image quality, and practices of the provider on an ongoing basis. Frameworks such as these may form a blueprint for assuring the quality of other radiology AI applications.

Feng et al. [6] propose an alternative methodology for monitoring AI algorithms using concepts such as statistical process control which are typically employed in industrial manufacturing but can also be used to support quality improvement in healthcare. “Special-cause” variation may cause unexpected changes in AI model performance and this can be tracked by looking at changes in the input and target variables and the relationship between them. Applying statistical limits to define a normal range will then help determine what is considered a breach and thereafter be subject to root cause analysis and correction.

Using natural language processing (NLP) to extract clinical information from radiology reports [23] may provide a more automated and efficient method of comparing clinical results against AI prediction. This could potentially reduce the resource costs needed for a human to review every individual radiology report to determine a ground truth diagnosis.

The ability to monitor for drift using an independent reference ground truth provides an alternative to using the primary radiology report which often serves as a proxy for ground truth despite its limitations [24]. Roschewitz et al. [25] demonstrated automated recalibration and tracking of changes to prediction distribution caused by shifts in acquisition inputs on mammograms. Venugopal et al. [26] similarly spoke of “temporal divergence” as a method to monitor the change in predictions made by an algorithm over time as well as also proposing the concept of “predictive divergence” which compares the prediction of the deployed model against two similar supplementary AI models to highlight any discordance. Merkow et al. [27] showcased a multi-modal approach to detect drift on chest radiographs using the inputs, image appearance representation, imaging metadata, and model output predictions. Sandvig et al. [18] captures these different monitoring methods in their scoping review of monitoring AI in healthcare.

The governance structures proposed by early adopters [17, 28, 29] provide recommendations on workflow-level designs for monitoring AI practically, e.g., through live interactive user feedback or retrospectively through auditing previous studies [21]. Becker et al. [30] conducted a survey in 2022 which showed that 58.4% of 185 respondents were not comparing the AI diagnostic accuracy against the radiologist diagnosis. It is, however, still not entirely clear how most users are monitoring their radiology AI applications today and what challenges they face in doing so [18].

A statement from multiple international radiology societies [24] has acknowledged the difficulties associated with monitoring imaging AI such as how to derive accurate and contemporaneous ground truth or how to monitor applications which perform quantitative tasks that are not easily measured by humans. Several suggestions to improve AI monitoring were made including a preference for real-time continuous monitoring instead of periodic monitoring (yearly re-evaluation of AI models in clinical use is recommended as a minimum); the formation of AI governance groups which can define processes for monitoring, escalating, and resolving problems related to AI deployment; and the creation of registries which can track the deployment of AI geographically.

### Regulations and Best Practice

The manufacturer requirements for post-market surveillance (PMS) are stated by the Food and Drug Administration [31], in the EU Medical Device Regulations 745/2017 [32], and within the recommended ISO 13485:2016 standard for quality management systems [33]. Collaborative work towards the harmonization of standards can also be seen in the AI/ML working group of the International Medical Device Regulators Forum which is building on the principles of Good Machine Learning Practice that considers AI applications from a Total Product Life Cycle perspective and includes provisions for predetermined change control plans [34, 35].

For AI adopters and healthcare providers, there are numerous standards and frameworks which state the importance of AI monitoring such as the BS 30440 framework for an AI management system [36] and the National Institute of Clinical Excellence Evidence Standards Framework for digital health technologies [37]. In the USA, the American College of Radiology (ACR) has devised a set of guidelines to help ensure radiology facilities can adopt AI safely and effectively as part of their national ACR Recognized Center for Healthcare-AI (ARCH-AI) program [38]. The Coalition for Health AI (CHAI) is a large ecosystem of academic health systems and organizations also looking to establish best practices for health AI; they have recently released a draft assurance standards guide [39] which again takes an AI lifecycle approach including the monitoring of the AI application. Interestingly, CHAI is also proposing a network of AI assurance labs to evaluate AI models. While these are not exhaustive examples of published standards, the practicalities and specifics of how these AI models should be monitored in a live radiology department are not often covered.

The establishment of registries to record where radiology AI applications have been deployed can help both improve transparency and aggregate real-world data which can support post-market surveillance and monitoring [24, 38, 40]. The UK Royal College of Radiologists AI registry [41] is now live and tracking AI deployments in the UK on a voluntary basis (it currently does not capture performance data). The ACR has also recently launched ‘Assess-AI’, an AI quality registry intended to monitor the performance of imaging AI applications deployed across the USA [42].

Previous studies have qualitatively explored concepts of clinical AI implementation and quality assurance with relevant stakeholders [43–45]. This study will explore specifically how radiologists are monitoring radiology AI applications given that they compose over 76% of FDA-cleared AI-enabled medical devices [1] and are relatively more commercially mature than AI for other non-imaging clinical specialties. It will also look at what barriers radiologists are facing when monitoring AI and how this could be improved. It is hoped this will help build awareness and bridge the gap between theory and safe practical implementation of radiology AI.

## Methods

### Interview Sample

Thirty-six potential interview candidates across the USA and Europe of varying levels of experience and working in different organizational structures were identified by authors JC and RL. The purposive sample was comprised of radiologists from the USA and Europe all of whom had experience or an interest in AI in healthcare.

The inclusion criteria for selection were practicing radiologists with previous experience or interest in radiology AI which may have been evident from their previous publications, presentations, or thought leadership on professional networking platforms such as LinkedIn. Non-radiologists were excluded from the sample in order to maintain homogeneity in the study population; however, geographic breadth of opinion was sought by identifying candidates in the USA and Europe.

The identified candidates were contacted via LinkedIn or email. The invitation to all participants included an introduction to the aims of the study and the option to schedule a virtual interview if the participant was interested. Of the initial 36 potential interview candidates, 3 had declined and 17 had not responded leaving a total of 16 candidates who proceeded to interview.

### Setting

A qualitative semi-structured interview study design was selected as the preferred methodology as it facilitates the exploration of radiologist perceptions and subjective experiences while also providing the opportunity to engage in open conversation [46].

The interviews were conducted by JC and lasted between 30 and 60 minutes depending on the availability of the participant. All participants were interviewed virtually over Microsoft Teams with the exception of one who was interviewed over the phone due to connection issues. Interviews were conducted between April and June 2024. The interviews were recorded with the consent of the participant and automatically transcribed using Microsoft Teams. The participant who was interviewed over the phone was not recorded; however JC, documented and wrote notes on the content of the call after it had finished. To preserve privacy, identifiers were removed during the storage of the recording. The recordings and transcripts were deleted upon completion of the study.

The questions were semi-structured and based on a topic guide (Table 1). The topic guide is based on considerations for AI monitoring identified by the literature [24]. Data saturation was obtained by the time the last participant had been interviewed, and no further new insights were being drawn.

**Table 1:** Interview topic guide

Introduction	Study overview Participant background & experience
Current practice of AI monitoring	What AI applications have been or are currently deployed at interviewees site of work Interviewee views on AI governance How does interviewee currently monitor AI
Thoughts on AI monitoring best practice	Views on important metrics Views on different methods of monitoring (e.g. manual vs automated) What are the biggest difficulties when monitoring radiology AI
Recommendations	Suggestions on how to improve AI monitoring

The process of interviews was iterative and adaptive so that slightly different questions could be asked in successive interviews to uncover additional insights.

Out of the 16 participants, the interviewer had previously known 9 of them in a professional capacity. The interviewer shares similar characteristics with the study population having previous clinical experience as a radiologist and having worked in a radiology AI company at the time the study was being conducted. While this helped facilitate peer-to-peer interaction and the usage of common terms when describing thoughts and experiences with AI, the interviewer clarified the research study was being conducted in his capacity as a UCL Master’s student and that the findings would be publicly shared.

### Ethics

No patients were involved in the study. Interview participants are not individually identifiable by any particular quote or opinion. The participants were informed of the purpose of the study before the virtual meeting and given the choice to decline. Interview participants reserved the right to withdraw from the study at any point. The study has been processed through UCL’s ethics review process.

### Analysis

The interviews were automatically transcribed by Microsoft Teams software and subsequently uploaded to NVivo qualitative data management software. In places where there was a transcription error, JC reviewed the recording and corrected the transcription. Inductive thematic analysis was undertaken by JC focusing on the explicitly expressed meaning of the data as expressed by Braun and Clarke [47]. This involved reviewing the transcripts to facilitate familiarization of the data and formulation of the initial set of descriptive codes [48] relevant to the research question. These codes were then clustered to form the core themes. Analysis occurred in parallel to data collection which allowed exploration of novel ideas in successive interviews until saturation. Interview participants did not review the transcripts or provide feedback on findings.

## Results

Six hundred minutes of interviews across 10 Europe and 6 USA-based radiologists (Table 2) yielded 32 codes which were categorized into 6 overlying themes as shown in Table 3. The majority of radiologists had between 10 and 19 years of clinical experience. The three most popular use cases were AI to support the detection of abnormal lesions on mammograms (8 counts), AI to analyze chest X-ray for abnormalities (8 counts), and AI to detect lung nodules on chest CT imaging (5 counts).

**Table 2:** Interview participants

Participant ID	Years of radiology experience	Region	AI use cases (previous and current)
P1	10–19	Europe	Mammography, chest X-ray analysis, stroke detection
P2	10–19	Europe	Mammography, chest X-ray analysis
P3	30–39	Europe	Mammography, chest X-ray analysis, CT lung nodule detection, prostate analysis
P4	0–10	Europe	CT head analysis, CT lung nodule detection
P5	10–19	USA	Mammography, image enhancement, quality control of vascular findings on CT, CT triage of acute findings, fracture detection
P6	10–19	USA	Mammography, MRI spine assessment
P7	10–19	Europe	Fracture detection
P8	10–19	Europe	Chest X-ray analysis, stroke detection, cardiac segmentation
P9	20–29	USA	CT lung nodule detection, chest X-ray analysis
P10	10–19	Europe	Bone age, cardiac CT
P11	30–39	USA	CT lung nodule detection
P12	10–19	USA	Mammography
P13	10–19	Europe	Chest X-ray analysis
P14	10–19	Europe	Mammogram, prostate analysis, bone age, CT lung nodule detection, stroke detection, chest X-ray analysis
P15	20–29	USA	-
P16	0–10	Europe	Mammography, chest X-ray analysis, stroke detection

**Table 3:** Summary of findings showing the core themes and constituent codes

Theme	Codes	Explanation
Current practices of monitoring radiology AI	Manual retrospective review	Manual comparison between radiology report (taken as ground truth) and AI output retrospectively to assess model accuracy
	Users flagging interesting cases	Users manually record discrepant or interesting cases for further review and discussion with the AI committee/vendor
	Surveys	Customised surveys were sent to relevant stakeholders to gather qualitative feedback
	Human-in-the-loop measurement validation	Human validation of AI outputs after image processing with editing as necessary
	Natural language processing (NLP)	AI technique that can parse textual data in radiology reports to extract the diagnosis for comparison against the AI results
Governance groups and who should be involved	Heterogenous maturity of governance groups	Different levels of maturity, readiness, and constitution of AI governance groups
	Inter-organizational governance	Different organizations may have their own AI policies and priorities which may not always align locally
	Stakeholders	Diverse representation to include medical physicists, radiographers, technologists, residents, and patient representatives
What metrics should be monitored	AI model accuracy	Measure of AI model performance using metrics such as F1 score, sensitivity, specificity, positive predictive value, negative predictive value, concordance, and discordance
	A priori model assessment	Measuring AI input and output independent of ground truth to look for the model shift, e.g., change in distribution or frequency of AI model predictions
	AI model explainability	Assessment of how interpretable the AI model is at deriving its predictions
	AI model bias	Monitoring for systematic AI errors that unfairly disadvantage certain groups or individuals
	Qualitative feedback	Gathering feedback on AI impact which may be difficult to quantify
	Broader patient outcomes	Monitoring the downstream impact of AI on the patient’s management and outcomes
Difficulties faced	Lack of allocated time and resources	Insufficient provision of dedicated time and recognition of stakeholders involved in AI projects can hamper progress
	Lack of compliance	Engaging users to consistently record AI errors can be difficult as it can add to workload
	Poor data integrity	Imaging data may not always be complete, e.g., demographic data may not be wholly captured which can limit AI bias monitoring
	Lack of instruction from vendor	AI vendors did not always have the ability to meaningfully monitor their AI application or share clear processes to do so
	Information governance	Review and approval for data sharing can be slow and inefficient limiting the ability for the vendor to monitor AI
	Lack of education	Lack of awareness and formal education around managing all stages of AI project can impact confidence and the safe implementation of AI
	Uncertainty around NLP	NLP is relatively untested for the purpose of radiology AI monitoring. Further investigation is needed into the feasibility of this method, e.g. accuracy and robustness of NLP to extract diagnosis from textual data
Who bears responsibility	Radiologist has duty of care	Radiologists ultimately have the duty of care to the patient, particularly in the absence of fully autonomous radiology AI applications, and should therefore ensure any clinical decision-support tools do not cause harm to their patients
	Both vendor and radiologist share responsibilities	Vendors have regulatory obligations for post-market surveillance of their AI devices. They need to work together with healthcare providers to ensure AI device safety
	Compensation	Consideration should be made around appropriate incentives for healthcare providers who may be expending significant effort in gathering performance data for the vendor
Recommendations	Investment in resources	Appropriate provision of resources into AI projects including allocated time and personnel to create a robust multi-disciplinary team. Nominate a clinical champion to lead and guide the project
	Start and iterate	Start simple, for instance with the manual comparison of AI and the ground truth on a periodic basis. Iterate and improve processes over time
	Tailored frequency of monitoring based on use case	Assess the impact on patient safety of your implemented AI solution and tailor your frequency and monitoring framework appropriately
	Alternative methods of monitoring	Consider and utilize multiple methods of monitoring, e.g., qualitative feedback or statistical methods that do not require ground truth
	Education	Educate all impacted stakeholders on the benefits and limitations of AI Encourage users to share AI discrepancies and feedback to facilitate peer learning
	Imaging networks	Utilize regional networks to better leverage local resources and support small hospitals which may be more resource constrained
	National organizations	Refer to guidelines from national professional organizations to aim for standardization of processes across the country

### Current Practices of Monitoring Radiology AI

Ten of the participants are currently not monitoring AI because many of their AI projects had been in an early validation phase and had not yet been widely implemented into regular clinical practice. Most responses were therefore capturing how they performed in their initial evaluation of the AI model and using that as a basis of how to potentially monitor on an ongoing basis. Several methodologies emerged:

A process of manual retrospective review that involved comparing the AI results against the radiology reports was found to be a fairly common approach given it is relatively simple to perform despite being time-consuming. Participants provided examples of performing retrospective evaluations of chest X-ray AI applications, chest CT, and CT head algorithms in such a way. Another participant similarly performed a retrospective analysis of chest X-ray AI performance; however, the radiologists were instructed to apply a designated structured code when reporting their chest X-rays which would allow for easier comparison between the radiologist interpretation and the AI prediction.

Several different options were identified which allowed the radiologist to save and flag incorrect or interesting AI cases for further review and feedback. These included setting up a secure File Transfer Protocol where anonymized studies could be uploaded and sent to the AI vendor for feedback, setting up a separate folder or worklist within the PACS where radiologists could easily save AI cases to review with the AI team, and also using QR codes which would allow radiologists to provide feedback via a structured form to the local AI leads. The local AI leads would have allocated time within their job plan to periodically review all flagged cases and subsequently educate all users of what may be causing the AI models to fault. This feedback is also fed back to the vendor to support post-market surveillance activities.

Two participants (P3, P13) employed the use of customized surveys as a way to gather qualitative feedback on AI from a range of different stakeholders including referring clinicians and patients. These surveys could be based on key metrics important to the AI adopter and sent out at different time points during the project lifecycle to assess the before and after impact of deploying AI.

The breadth of AI applications available extends beyond triage and detection of abnormalities to segmenting and quantifying different anatomical structures, for example, measuring the cardiac contours or spinal alignment on MRI imaging. In these instances, participants described a human-in-the-loop scenario whereby a healthcare professional would review the measurements for accuracy and make adjustments as necessary. Adjustments are recorded and followed up for discussion in local AI governance meetings.

The aforementioned monitoring methods require manual human effort which can be a resource constraint amongst healthcare providers. One radiologist in the USA described using natural language processing on radiology reports to derive the ground truth on which to compare against AI. This would allow for monitoring at scale while also producing a list of discordant cases which would be subject to further human review. While it was acknowledged that NLP may not be accurate all of the time, with large enough volumes, it may be sufficient to reduce statistical noise and provide a gauge of AI to human congruence.

### Governance Groups and Who Should Be Involved

AI governance groups or steering committees varied depending on the type of organization in which the radiologist worked and the maturity of their AI strategy. Participants noted how the core responsibilities of these groups included reviewing project proposals and discussing AI use cases and implementation processes.

Interestingly, within the UK, there may be multiple different healthcare organizations which overlap, such as individual NHS trusts and regional imaging networks, but have their own different AI strategies and governance structures. This may sometimes cause confusion:

"Many trusts still don’t have AI committees. They don’t know what the committee means and who should be on it." (P13)

A similar sentiment was echoed by participant 2 who believed AI governance should be done at the network level so that individual trusts are not pressured or overstretched and existing expertise can be better utilized across the whole network.

While a top-down approach may be advantageous for ensuring alignment, it can also be challenging when decisions are separated from the specific needs of local radiology departments:

"Directives going through the integrated care systems may not necessarily match up with what is useful at a trust level." (P8)

Medical physicists were identified as useful members of the AI groups to help with the monitoring and quality assurance of AI applications given their pre-existing responsibilities which include ensuring medical imaging equipment is functioning safely. The medical device safety officer was also identified as having an important role in recording and overseeing AI medical devices in use locally (P4).

Two participants believed that patients should also be represented in AI focus groups as should radiology residents who may benefit from being educated around new AI implementations and supporting AI monitoring activities.

### What Metrics Should Be Monitored

Study participants were asked what metrics they believed were important to measure and monitor. Many participants recognized that measuring basic AI model metrics such as accuracy, sensitivity, and specificity is important. In the first instance, these could be compared over time against the initial baseline AI performance results to identify model drift.

One participant highlighted it would be useful to monitor AI models in an “a priori” fashion without needing to rely on an external comparator or ground truth. This could be done by looking intrinsically within the imaging metadata or distribution of predicted model outputs [27] to highlight statistical variation.

Another participant made the point that it is important to be able to monitor how the AI model is deriving its outputs to provide enhanced AI explainability. In the context of AI applications that segment and measure anatomical regions such as the brain:

"I don’t think it’s our role to be validating the actual measurement. Our role is to validate the way that it is performing the measurement. Are the highlights correct and are they fitting in the right anatomy?" (P5)

Performance across different patient demographics and subgroups was noted as being important to monitor given the potential for AI applications to exacerbate health inequality [49]. One participant also believed that greater transparency in what datasets were used to train the AI model would help build trust and awareness around potential AI model bias.

A more holistic assessment of the AI impact could be ascertained by gathering feedback from a range of different affected stakeholders. This would provide qualitative insights rather than solely relying on quantitative data which may fail to capture the complete picture. AI is often used as a tool to help address a problem; it is therefore important to measure the success criteria of the original problem and contextualize the broader workflow in which the AI will be placed in order to properly appreciate the patient impact:

"Monitor against the objectives of the project… the clinical benefits and the impact on the workforce etc. For this chest x-ray AI application, we’re monitoring the turn-around-times for patients and the impact that the AI has on prioritising these patients (for expediting appropriate patients onto a lung cancer screening pathway)" (P3)

### Difficulties Faced

A broad range of challenges was elicited by all the participants that were not localized to any one particular region. The most common sentiment amongst participants was a lack of allocated time and resources which made it difficult to manage and evaluate an AI project. There were variations amongst the participants interviewed with some having dedicated time within their job plan to focus on AI initiatives, whereas for others, it was not formally accounted for in their job plan:

"It is time intensive and requires engagement from clinicians as well as other stakeholders which is difficult within an already overstretched workforce." (P14)

"It is important to have funds, resources and time for people to do it… I had to do it voluntarily without getting paid for this work or even getting time recognised." (P13)

Several participants reported that getting all users to consistently record or document AI errors can be difficult due to a lack of incentive and the extra workload it can create. This includes scenarios with in-workflow tooling such as a dedicated button on the screen which radiologists can click to record whether they agree or disagree with the AI results.

"It’s all very person dependent, some people feel motivated to do that, some don’t have time for it." (P13)

"Some people say monitoring is just having a button to say agree or disagree (with the AI results), that’s not monitoring, that’s alert reporting because it depends on the user clicking a button … it leads to a sort of bias because some will click, some won’t click and you often click the errors rather than the normals." (P1)

"One of the selling points of AI is to reduce the radiologist burden. If you now ask them to click a button every time then it doesn’t really serve that purpose." (P4)

Participants acknowledged the risk of AI bias but also saw that data integrity can be a challenge as demographic information, such as patient ethnicity, may not be well documented which can make monitoring for bias more difficult.

"Demographics is from my experience quite poorly captured in metadata so how do you actually track that accurately?" (P15)

While vendors of AI software have a regulatory duty to conduct post-market surveillance of their commercial products, there was variability in how much vendors supported the AI adopters in their monitoring process:

"We were offered no monitoring suggestions by the vendor…the vendor did not set up a feedback mechanism." (P11)

Two participants considered information governance to be a challenge in AI projects. Vendors may require access to patient data to support monitoring; however, the healthcare provider may have information governance policies in place which restrict access.

"Biggest challenge in actual implementation of monitoring is to find a consistent way of getting agreement between information governance and the DPIA over what data can be shared.” (P1)

The lack of a consistent process around how to implement AI from start to finish was picked up by another participant who emphasized how a lack of education around AI acted as a barrier to engagement and deployment.

"The biggest problem I see is actually getting any AI tools in there. There’s a lot of reluctance because of all of these different challenges that people don’t know how to monitor or what the implications are, or even how to get it onto their system." (P10)

Once implemented, there was a lack of clarity over what defines an “AI adverse event” and what the appropriate escalation procedures should be. This is in contrast to pharmaceuticals in clinical care where there is an established process to report adverse events and raise concerns to protect patient safety.

"We need to define what an AI adverse event is and once defined we have to set up a reporting system so that I know how to report it to the vendor." (P11)

When discussing the different monitoring methodologies, there was a degree of skepticism over the more automated techniques. NLP, for example, can help extract the ground truth or diagnosis from a radiology report; however, this can be inaccurate as the radiology reports may be non-standardized in terminology and at times ambiguous in their assertion of a diagnosis.

"I don’t know if right now the techniques are strong enough or established yet so if you’re going to monitor with that you have two variables." (P14).

"Should we trust the NLP tool? There’s not much evidence we can find on it at the moment so how did you validate your NLP and how much uncertainty does it add?" (P16)

### Who Bears Responsibility?

The general sentiment amongst interview participants was that the responsibility for monitoring AI should be shared between both the vendor and the healthcare provider. Several of the participants leaned towards the user/healthcare providers having most of the responsibility as they hold the primary duty of care to the patient.

"I don’t think we can leave it just to the vendors to mark their own homework … I think it’s really important for the users to take some responsibility because if the goal of using AI is to optimise patient care and a model is not performing well, we’re neglecting that basic duty ."(P16)

Given the resource cost and radiologist expertise that is needed to feedback and monitor these AI algorithms, there was a question of whether the healthcare provider should be compensated in some way for providing this valuable information to a private vendor. Two participants believed compensation would be fair and could take several forms such as reimbursement, product discount, paid research time, or provision of resources such as a medical writer if there were plans for publication.

### Recommendations

Participants were asked an open-ended question on what suggestions or recommendations they had that would improve the process of AI monitoring today. Given that a lack of resources was a common problem, a solution would be to further invest in resources to implement and monitor AI: A clinical champion or AI lead would significantly help ensure project success and provisions should be made within their job plan to reflect their ownership of the project.

"The only way that you can deliver an AI project is to have a champion and to have somebody who’s going to push it forward… because the amount of work is unbelievable." (P8)

There was recognition amongst participants that many questions on how to monitor AI do not yet have clear answers given there is not yet widespread radiology AI in routine clinical use. Pragmatism should be prioritised so that adopters can begin learning and sharing their experiences to build best practices and solutions.

"I think people generally accept that there is no set way but we just need to start with something." (P1)

The frequency of monitoring should be dependent on the AI use case and its potential impact on patient care. Critical AI applications, such as stroke management, would necessitate much more regular monitoring. Any changes in hardware or software should also be a trigger to re-check the performance of the AI algorithm. This could all initially be done manually but moving to an automated system would allow for scalability in the long run.

Four participants thought alternative methods of monitoring which do not rely on the presence of a ground truth could be considered a supplemental monitoring methodology. Incorporating patient and clinician feedback was also suggested to provide more holistic information on the value of the AI application over time.

Education was seen as a crucial component for successful change management and engagement. By regularly capturing feedback and monitoring AI performance, any errors can be shared with other users which can help set the right expectations and build awareness around the benefits and limitations of AI algorithms.

"The more you give them feedback on what has happened, the more they are incentivised to give more, otherwise it just fades off and they go back to their routine. So what I learned from my experience is that regular feedback is a key feature of change management to keep them engaged. If you just tell them once or twice they’ll forget." (P7)

A UK-based participant believed imaging networks [50] could play a lead role in supporting joint procurements and enabling collaboration by efficiently leveraging regional expertise. Imaging networks could also facilitate the creation of ground truth datasets that can be used for regular testing of AI models. Another participant believed national professional organizations such as the Royal College of Radiologists in the UK have a role to play in aligning policy and providing standardized guidance on monitoring.

## Discussion

The primary aim of this research was to better understand how AI in radiology is currently being monitored today and what difficulties radiologists face in doing so.

The 16 participants represented a broad geographic breadth with the majority (10) having between 10 and 19 years of radiology experience. Although there were references to UK-specific institutions such as NHS imaging networks, there was not a significant difference in the type of barriers and difficulties faced by the radiologists in the USA and Europe with resource constraints being a universal issue.

Interestingly, the chest X-ray AI use case was over-represented (7 counts) in Europe and only 1 count in the USA suggesting differences in use case adoption. This may be accounted for by different regional clinical pathways; in the UK, the National Optimal Lung Cancer Pathway (NOLCP) [51] recommends that primary care referred chest X-rays are reported within 24 hours, and if an abnormality is found that is suspicious of lung cancer, that patient should then go on to have CT imaging within 3 days. AI for analyzing chest X-rays is being explored by UK healthcare providers as a way to meet NOLCP recommendations and expedite lung cancer diagnosis [52]. Important metrics to monitor could therefore be dependent on the particular pathway they are intended to improve, e.g. is AI improving compliance with NOLCP targets and is there a downstream trend of improvement in patient diagnoses and outcomes? Another divergent workflow relates to mammography interpretation, where in the US, this is typically done by a “single reader” (one human reader and typically a computer-aided detection system), but in Europe, it is more common to use a “double reader” (two human readers) workflow [53]. This may again have implications on how specific AI use cases are regionally monitored as they will impact workflows and patients in different ways.

It is also important to bear in mind that there may be regional differences in what AI features are commercially available between different regulatory jurisdictions [54]. CE-marked chest X-ray AI applications, for example, tend to have a broader feature set than their FDA counterparts by generally allowing for the identification and localization of multiple imaging abnormalities whereas the FDA version may only allow for the triage of a much smaller range of findings. This may affect the regional adoption rates of different use cases and explain the discrepancy in chest X-ray AI popularity between Europe and USA. It may also be relevant if considering harmonizing data registries [24] between different countries to track and record AI deployments [42].

Ten of the sixteen participants were not currently monitoring AI as their projects had been in an evaluation phase rather than a full-scale live implementation. This paucity in mature radiology AI implementations aligns with findings from previous studies [18, 55] and may help explain why ongoing AI monitoring is not yet widely done [30].

The most common monitoring methodology involved a manual process of retrospectively reviewing AI-processed studies alongside the reported findings despite this being resource-intensive to perform at scale. Much of the experiences doing this were also in the pre-deployment evaluation phase as opposed to on an ongoing basis. In only one case, natural language processing was being employed to extract information from radiology reports. The study findings show that it tends to be the more clinically focused AI methodologies mentioned in the literature [17, 18, 24, 55], such as review of AI-human discordances or human interaction to verify AI correctness, that are being employed. In contrast, there is a lack of more statistical monitoring methods being utilized amongst participants, such as feature importance, target variable, or input monitoring [18]. This may be because the study sample consisted exclusively of clinical radiologists but it may also suggest that statistical methods of AI monitoring may require non-clinical expertise which is not yet adequately represented in AI governance groups.

Anderson et al. [18] identified 5 performance metrics to measure from their scoping review: accuracy, discrimination, calibration, proxy outcomes, and fairness. Accuracy and proxy outcomes were well-represented responses in this study sample as was monitoring for fairness (model bias) although this was recognized as difficult to perform in practice. Zhang et al. [56] identified similar and additional machine learning metrics: correctness, model relevance, robustness, security, efficiency, fairness, interpretability, and privacy. Technical considerations such as efficiency (inference time) and security were not routinely mentioned amongst study participants which again highlights the importance and utility of a multi-disciplinary team to consider all pertinent metrics. Financial considerations were not discussed but may be important to monitor to ensure that procured AI applications demonstrate ongoing monetary value.

The difficulties reported by study participants stressed the importance of appropriate resource and time allocation to perform AI monitoring. Other challenges including information governance and lack of staff education around AI have been commonly reported in other studies [44, 57, 58]. Through a multi-disciplinary cross-sectional survey exploring medical imaging AI governance, Stogiannos et al. [29] found that the top priority for successful AI adoption was guidance or standards on AI validation and evaluation.

The idea of having a specified group of stakeholders to oversee AI projects resonated with all participants and is well recognized as important for AI adoption [29]. There was recognition of the role multi-disciplinary team members could play [59], in particular medical physicists, radiographers/radiologic technologists, and patient representatives, in these AI committees. AI vendors themselves were also seen as important stakeholders in guiding AI monitoring efforts and could work towards technically supporting the data flow in the process, e.g., capturing performance data in the background which could then be automatically fed into relevant national registries [17]. Going forward, collaboration and communication with vendors may grow in importance as the FDA has now finalized its guidance on Predetermined Change Control Plans (PCCP) for devices utilizing AI-enabled software functions [35]. This will allow AI vendors to modify their AI device post-deployment in accordance with the modification protocol set out in a previously authorized PCCP, without requiring a new marketing submission. This process may help realize the iterative benefits of AI technology more efficiently and potentially help improve local AI model performance.

### Limitations

The study was limited to 16 radiologists only; a broader range of cross-disciplinary stakeholders, e.g., involving AI developers, radiographers, regulators, patient representatives, and IT administrators etc. would have provided a variety of opinions and potentially a more holistic picture of the challenges faced when monitoring radiology AI.

While geographic diversity was sought, there are many country-specific healthcare organizations in Europe which may have their own processes for AI monitoring which are not captured here and for which the findings may not generalize.

Only one researcher conducted the qualitative analysis and derived the underlying themes from the codes as part of his dissertation research. A second reviewer may have identified different themes from the data.

The majority of current commercially available radiology AI applications [1] are based on convolutional neural networks for image recognition. Newer generalist AI foundation models can interpret multi-modal data [60] and generate textual content as an output. These models had not yet been used by the interview participants and subsequently were not discussed. Further research is recommended on how to monitor large language models and foundation models within radiology as they can present their own unique set of challenges and considerations.

## Conclusion

This qualitative interview study of 16 radiologists suggests that mature deployments of radiology AI applications remain limited with few actively at the stage of employing robust monitoring processes. Manual AI monitoring methods, such as periodic retrospective reviews, were popular amongst participants; however, there was a relative paucity of automated and statistical methods of monitoring in use. Other challenges included a lack of resources and a lack of educational awareness of best practice guidelines.

A clear AI governance framework with a robust and well-represented multi-disciplinary team is essential to facilitate change management and ensure AI is monitored across multiple relevant domain metrics.

As radiology AI applications increase in adoption and maturity, it will be imperative that an effective quality control process accompanies their deployments. It is hoped that the sharing of experiences and learnings by AI adopters will support the ongoing development of tools and practical processes for safely monitoring radiology AI.
